# Antimicrobial Resistance (AMR)

**DOI:** 10.3389/bjbs.2023.11387

**Published:** 2023-06-28

**Authors:** Ka Wah Kelly Tang, Beverley C. Millar, John E. Moore

**Affiliations:** ^1^ School of Biomedical Sciences, Ulster University, Coleraine, United Kingdom; ^2^ Laboratory for Disinfection and Pathogen Elimination Studies, Northern Ireland Public Health Laboratory, Belfast City Hospital, Belfast, United Kingdom

**Keywords:** infection, AMR, antibiotic resistance, antimicrobial resistance, one health

## Abstract

Antimicrobial resistance (AMR) has now emerged as a chronic public health problem globally, with the forecast of 10 million deaths per year globally by 2050. AMR occurs when viruses, bacteria, fungi and parasites do not respond to antimicrobial treatments in humans and animals, thus allowing the survival of the microorganism within the host. The prominent cause contributing to the current crisis remains to be the overuse and misuse of antimicrobials, particularly the inappropriate usage of antibiotics, increasing the global burden of antimicrobial resistance. The global consumption and usage of antibiotics are therefore closely monitored at all times. This review provides a current overview of the implications of strategies used by international governmental organisations, including the UN’s 17 Sustainable Development Goals (SDGs), to address the problem of antibiotic resistance, as well as the “*One Health Approach*,” a system incorporating a multidisciplinary effort to achieve the best possible health outcome by acknowledging the clear connections between humans, animals and their shared environment. The importance of public awareness and health literacy of lay audiences still needs to be further emphasised as part of global and local action plans. Antimicrobial resistance continues to be a major global public health dilemma of the 21st century. Already this topic is receiving substantial political input from the G7 countries and continues to be on the agenda of numerous political conferences. The consequences of failure to adequately address AMR are profound, with estimations of a return to the pre-antibiotic era, where everyday infections relating to childbirth, surgery and open fractured limbs could be potentially life-threatening. AMR itself represents a microcosm of factors, including social anthropology, civil unrest/war, diasporas, ethnic displacement, political systems, healthcare, economics, societal behaviour both at a population and individual level, health literacy, geoclimatic events, global travel and pharmaceutical innovation and investment, thus finding a solution that adequately addresses AMR and which helps stem further AMR emergence is complicated. Success will involve individuals, communities and nations all working together to ensure that the world continues to possess a sufficient armamentarium of effective antimicrobials that will sustain human and animal health, both now and in the future.

## Introduction

Antimicrobial Resistance (AMR) results when microorganisms including bacteria, fungi, parasites and viruses evolve to the extent that they eventually become resistant to the antimicrobial medications, such as antibiotics, which are used to treat such conditions [1]. AMR has now emerged to be one of the greatest global concerns in the 21st century due to the rapid growth of AMR infections rate and the lack of new antimicrobial medications being introduced to combat this issue [2]. One of the main causes of the current issue could be the consequences of overuse or irresponsible use of antibiotics in various situations, primarily in clinical treatment along with agricultural usage, animal healthcare and the food system [3]. AMR is widely referred to as the “*Silent Pandemic*” and is a problem where urgent action is needed immediately and should be managed more effectively and not be considered as a future situation [4].

Without preventative measures, it is estimated that by 2050, AMR could potentially become the world’s primary cause of death [5]. According to estimates provided globally, the number of deaths directly linked to AMR has risen to more than 1.2 million in 2019 and this is forecast to increase to approximately 10 million deaths per year by 2050, if insufficient action is taken to control AMR [5].

In response to AMR, several global health organisations and governments have taken action to combat this issue. The “*One Health Approach*” was introduced which requires a global collaborative effort to involve a number of different disciplines: the Food and Agriculture Organization of the United Nations (FAO) and the World Organization of Animal Health (OIE), to ensure that each agency works within its speciality and with other agencies to minimise the potential effects of AMR [6]. In addition, the World Health Organization (WHO) has established the Global Action Plan for managing AMR (GAP-AMR) followed by launching the Global Antimicrobial Resistance and Use Surveillance System (GLASS) to continuously close the existing knowledge gaps in order to achieve the goals of GAP-AMR programme [7].

Within most of these newly proposed measures, one of the most effective approaches is to increase public awareness of the AMR pandemic as a preventative strategy, thereby requiring good communication with all stakeholders [8]. In this review, the aim is to examine the various current global AMR management strategies, to aid its control.

**FIGURE 1:**
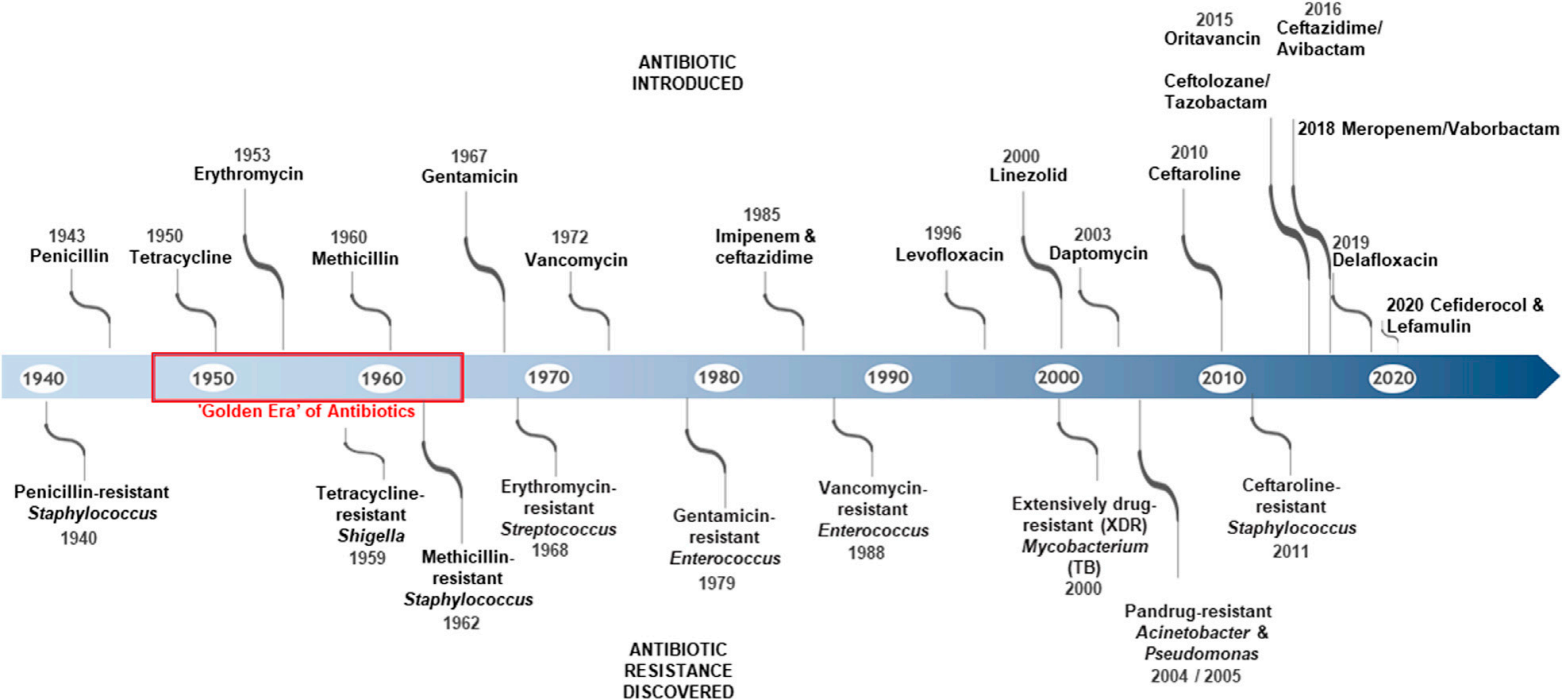
Timeline showing some of the key antibiotic discoveries and reports of the emergence of antibiotic resistance strains [12].

The creation of antimicrobials is one of the most effective drug treatments in medical history. The introduction of antimicrobials has helped to manage and greatly reduce death rates caused by infectious diseases, previously the leading cause of death in humans [9]. Human life expectancy has increased on average by 23 years, since the first antibiotic was introduced in 1910 [10]. With regard to that, the discovery of the antibiotic penicillin, by Sir Alexander Fleming was one of the greatest medical advancements of the 20th century which initiated the “golden era” of antibiotics [10]. However, shortly after, the production of penicillinase by antibiotic-resistant strains was reported, which led to inactivation of the antibiotic molecule, thereby rendering it clinically ineffective [11]. This is important to note, as penicillin and its derivatives (cephalosporins, carbepenems) are the major class of antibiotics still employed today, in treating human and animal infections. Over 150 new antibiotics have been developed since then and their widespread usage and overusage has led to the development of higher resistance and even multiresistant forms of the so-called “superbugs.” These have increased mortality rates due to a lack of clinical effectiveness, resulting from antibiotic use/misuse [11]. AMR is the term given to this resistance, which has been discovered in viruses, parasites, fungi, and bacteria. However, with regards to AMR, bacterial antibiotic resistance is particularly problematic due to its rapid rate of developing resistance to many new antibiotics, which are used to treat bacterial infections (Figure 1) [2].

## Epidemiology of AMR

Previous research has shown that one of the main causes of AMR is the inappropriate and excessive use of antibiotics, both in humans, as well as in animals. Since the 1950s, when the “golden era” of antibiotics began, Alexander Fleming had expressed concerns about the potential emergence of resistance if treatment is used for an inadequate amount of time [13]. “Since then, there has been an increasing demand for new and novel antibiotics, in order to combat highly resistant strains that have been emerging.”

As a result, the use of antibiotics in treating infectious diseases and other conditions has become widespread in modern medicine. Nevertheless, the misuse and even over-prescription of these antimicrobials has fast outpaced their efficacy as AMR has grown markedly [10].

A series of recent studies have indicated antimicrobial involvement in agriculture and food systems may have a significant impact on driving AMR [14]. According to estimates, in the US, 70% of antibiotics used to treat humans are available for use in veterinary medicine [15]. In recognising such high usage in global agriculture, several organisations, including the World Health Organization (WHO), the United Nations (UN) and the European Union (EU) have taken steps towards reducing and restricting the use of antimicrobials in animals, through the legislative banning of using certain antibiotics in agrifood systems for growth promotion, as well as promoting antimicrobial stewardship initiatives in treating food animals, as well as in small domestic companion animals. Such controls may be difficult to implement particularly in developing countries where the demands for food animals continue to rise annually [16].

In addition, zoonosis also poses a serious transmission factor of AMR, where antibiotic-resistant bacteria are spread between animals and humans, through either direct or indirect interaction, as well as through foodborne or waterborne events [17, 18].

A study conducted in China has reported that mediated colistin resistance (MCR-1) poses a high risk to AMR due to zoonosis transmission from animal to human [19]. According to this study, MCR-1 was detected in *E. coli* isolates from animal and commercial meat sources and MCR-1 is believed to have been widely transmitted in food-producing animals in south China. In contrast, relatively low MCR-1 was detected from human origin, this difference in the prevalence of MCR-1 between animals and humans is likely to be a form of zoonotic transmission from animals to humans [19].

Colistin is regarded as a “last resort” antibiotic that is increasingly being utilised to treat patients with multiresistant bacteria, although it is still effective it was not typically used to treat common infections due to its potentially adverse side effects. However, if colistin resistance evolves rapidly, these bacterial infections may be more difficult-to-treat [20].

The lack of knowledge of antimicrobials is also a major contributing factor to AMR. Public Health England conducted a UK survey in 2017 to find out public knowledge on antibiotics [21]. Eighty three percent of participants admitted that antibiotics could be used to treat bacteria and 35% of participants believed that antibiotics could be used to treat viral infections. These results have improved since the survey that was conducted in 2014, which indicates that the public in the UK has become more informed and educated about antibiotics [21]. In comparison to a survey conducted in India, it was found that 49% of the respondents stated that antibiotics could treat viral infections and 45% of respondents use antibiotics for treating colds [22]. As a consequence, India was reported as having one of the highest rates of infectious disease, including those caused by multi-resistant pathogens [23]. These studies indicate that there is a relationship between AMR and public awareness.

## Global Burden of AMR

With AMR emerging at a rapid rate, the infection and death rates of AMR are closely monitored at all times. In the United Kingdom, the estimated AMR infection rate was 65,162 people diagnosed in 2019, which increased from 61,946 patients recorded in 2018 [24]. In comparison, the European Centre for Disease Prevention and Control (ECDC) has reported in the EU alone, the infection rate of AMR has reached over 670,000 cases annually [25]. According to data analysis from a prior study, there were 4.95 million deaths worldwide in 2019 that were linked to bacterial AMR, and 1.27 million of those deaths were directly caused by bacterial AMR [26]. In a well-known review, it was previously reported that the annual death rate directly caused by AMR is predicted to rise to 10 million by 2050. With the highest estimated deaths of this being in Asia followed by Africa, mainly due large populations and lack of regulation associated with AMR prevention [5]. According to previous research, Sub-Saharan Africa has the highest all-age mortality rate in the Global Burden of Diseases (GBD) region that is directly linked to or related to AMR, in contrast to Australasia which had the lowest rate of AMR-related mortality in 2019 [26].

## Risk Factors Contributing to AMR in Specific Populations

Previous literature focusing on the different national responses to antibiotic resistance highlighted the key risk factors contributing to AMR between developing and developed countries and how each lead to AMR in a different manner. Several AMR contributing factors were found in developing countries which include, poor regulation control of antimicrobial drugs, insufficient monitoring of the emergence of AMR, inappropriate use of antibiotics in clinical settings and the inadequate quality check for antibiotics supplied [27].

Recent studies on the accessibility of antibiotics in low- and middle-income countries found that Vietnam and Bangladesh had the highest proportion of unlicensed locations where antibiotics are administered, with relevant antibiotics frequently found in typical drug stores for mild illnesses, where they are widely available to the general public [28]. Since those communities have such easy access to antibiotics, maintaining this attitude would lead to a number of issues, such as improper use of antibiotics due to a general lack of knowledge on antibiotics and awareness of AMR and with lack of consideration of the quality of the antibiotics distributed. All such factors may contribute to the emergence of AMR.

On the contrary, in developed countries, the risk factors that exist in developing countries may not necessarily apply. These include the excessive use of antimicrobials in agricultural use and over-prescription in clinical settings [27]. Research on this issue presents conflicting findings as a more recent study reported that China, as a developing country, became the top consumer of veterinary antimicrobials in 2017, accounting for up to 45% of global consumption and is estimated to remain the biggest user in 2030. In addition, with evidence that developed countries have reduced their total antimicrobial sales, for example, the UK had a 39.2% decrease from 2015 to 2017 [29]. These results suggested that risk variables in emerging countries are now beginning to overlap with those in developed countries.

## Global Consumption of Antibiotics

There is now a growing body of research that investigates global antibiotic consumption. The Global Research on Antimicrobial Resistance (GRAM) Project, conducted the first known long-term study to estimate the global consumption of antibiotics, which covered 204 nations from years 2000–2018. According to this estimation of the average daily dose, a significant increase of 46% in the global antibiotic consumption rate was observed throughout this period of time [30].

This seminal study through employment of a spatial disparities/geostatistical model, identified large national and subnational variations of antibiotic usage in Low- and Middle-income countries (LMICs), with the lowest levels in sub-Saharan Africa and the highest in eastern Europe and central Asia [30]. The study also showed a global antibiotic consumption rate of 14.3 (95% uncertainty interval 13.2–15.6) defined daily doses (DDD) per 1,000 population per day in 2018 (40.2 [37.2–43.7] billion DDD), an increase of 46% from 9.8 (9.2–10.5) DDD per 1,000 per day in 2000 [30]. There were large increases in the consumption of fluoroquinolones and third-generation cephalosporins in North Africa and Middle East and south Asia [30]. Carbapenem consumption was highest in the high-income region, where it increased from 0.05 to 0.09 DDD per 1,000 per day from 2000 to 2018 [30]. The surge in the misuse of antibiotics has remained the main factor in causing this trend and the emergence of AMR. The characteristic of inappropriate use of antibiotics includes using antibiotic for an insufficient amount of time than specified, treating conditions other than bacterial infection using antibiotics, as well incorrect administration methods and dosage taken [31].

Despite that, critics have also argued that this is not the only problem but an addition to issues of increased “last line antimicrobials” consumption. According to recent research, last-line antibiotics such as polymyxin consumption in humans has increased by 67% in the EU/EEA and these last-line antibiotics are typically prescribed in hospital settings to treat patients suffering from serious infections as a last resort to combat multidrug-resistant bacteria [32]. As a result, the increased consumption of such antimicrobials could eventually induce resistance and paradoxically make serious infectious diseases more difficult-to-treat.

This ongoing pattern has presented a strong correlation between AMR and antibiotic consumption. Several studies have supported this view, including a longitudinal study which reported a significant strong association between the use of antibiotics, particularly carbapenems and carbapenem resistance [33]. Similarly, this trend was supported by another study which also identified a strong correlation between the antibiotic consumed and the prevalence of carbapenem resistance in Gram-negative bacteria [34].

However, issues of the debate are a concern as previous studies have argued that a direct correlation may not be present between antibiotic consumption and AMR since other factors were ignored during the research. Many of these correlation studies were conducted in hospitals where patients are presented with underlying problems, the results of the correlation may be overstated [35]. Further research would be needed to be conducted in this area with a study being more inclusive and not restricted to a specific sector.

**FIGURE 2:**
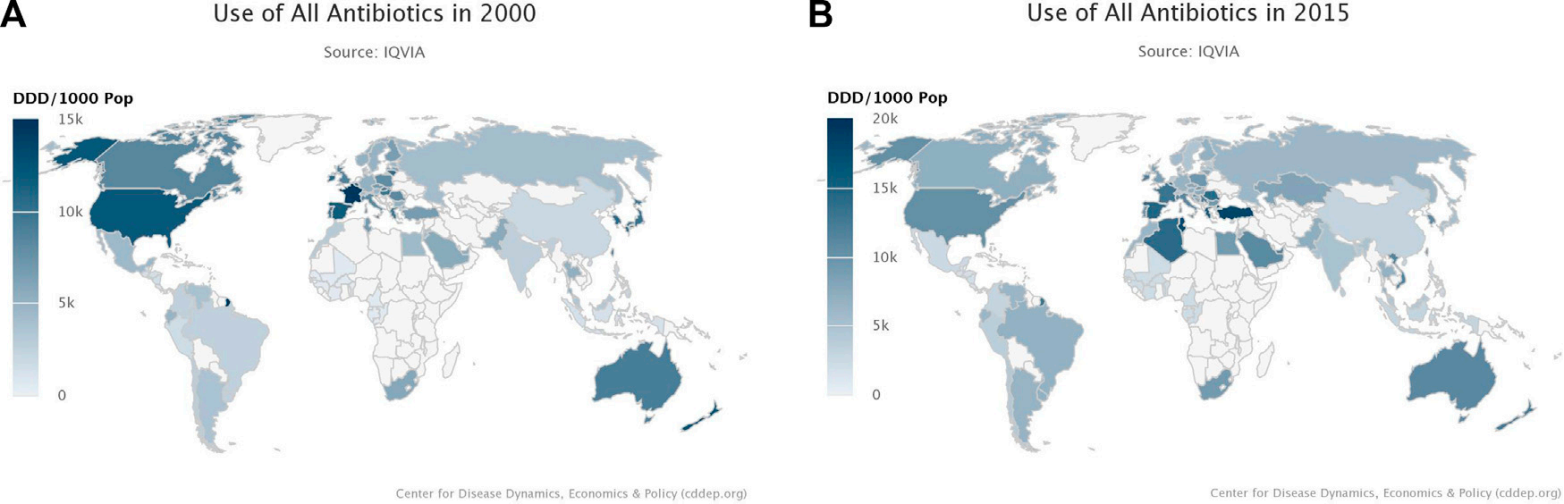
**(A)** Resistance Map showing the Global usage of antibiotics in 2000 [36]. **(B)** Resistance Map showing the Global usage of antibiotics in 2015 [36].

Antibiotic usage also influenced AMR in relation to the difference between LMICs and High Income Countries (HICs), resistance maps of global usage of antibiotics were collected worldwide from the years 2000–2015 (Figures 2A, B). A distinct pattern was observed, as seen in Figure 2A, when in 2000, the more developed continents including North America, Europe, and Australia were shown to have the highest overall antibiotics usage. However, compared to Figure 2B with data from 2015, it was observed that usage of antibiotic remains high in Europe, with a slight decrease in North America, in contrast, parts of Africa and Asia have been reported to have increased antibiotic use since 2000 [36].

Since there is evidence of a correlation between the overuse of antibiotics and the development of AMR, reducing antibiotics usage could be a beneficial goal in HICs, with over usage of antimicrobials. In contrast, increasing the accessibility of such antimicrobial medication remains a goal for many LMICs, as the death rate remains high caused by infectious diseases in many LMICs due to the lack of effective antimicrobials access to those in need [37]. In a study with children in Haiti, Kenya, Malawi, Namibia, Nepal, Senegal, Tanzania, and Uganda between May 2006 and December 2016, antibiotics were prescribed to 80.5% of children diagnosed with respiratory illness, 50.1% with diarrhoea, and 28.3% with malaria, where the mean number of antibiotic prescriptions issued to children between birth and age 5 years across the eight countries was 24.5 (95% CI 22.6–26.7), ranging from 7.1 (6.3–7.9) in Senegal to 59.1 (54.1–64.6) in Uganda [38]. The authors indicated that with such high prescription rates for antibiotics, that there was over-prescribing of antibiotics that needs addressing to avoid the emergence of AMR [38].

While there is ongoing research on new antimicrobial treatments, there must be controls and management of the antibiotics including standardisation of quality checks and accessibility of antibiotics. Therefore, improving surveillance programs is urgently required in some LMICs to improve the current state of AMR.

## What Global and Local Action is Being Taken to Address AMR?

### One Health Approach

**FIGURE 3:**
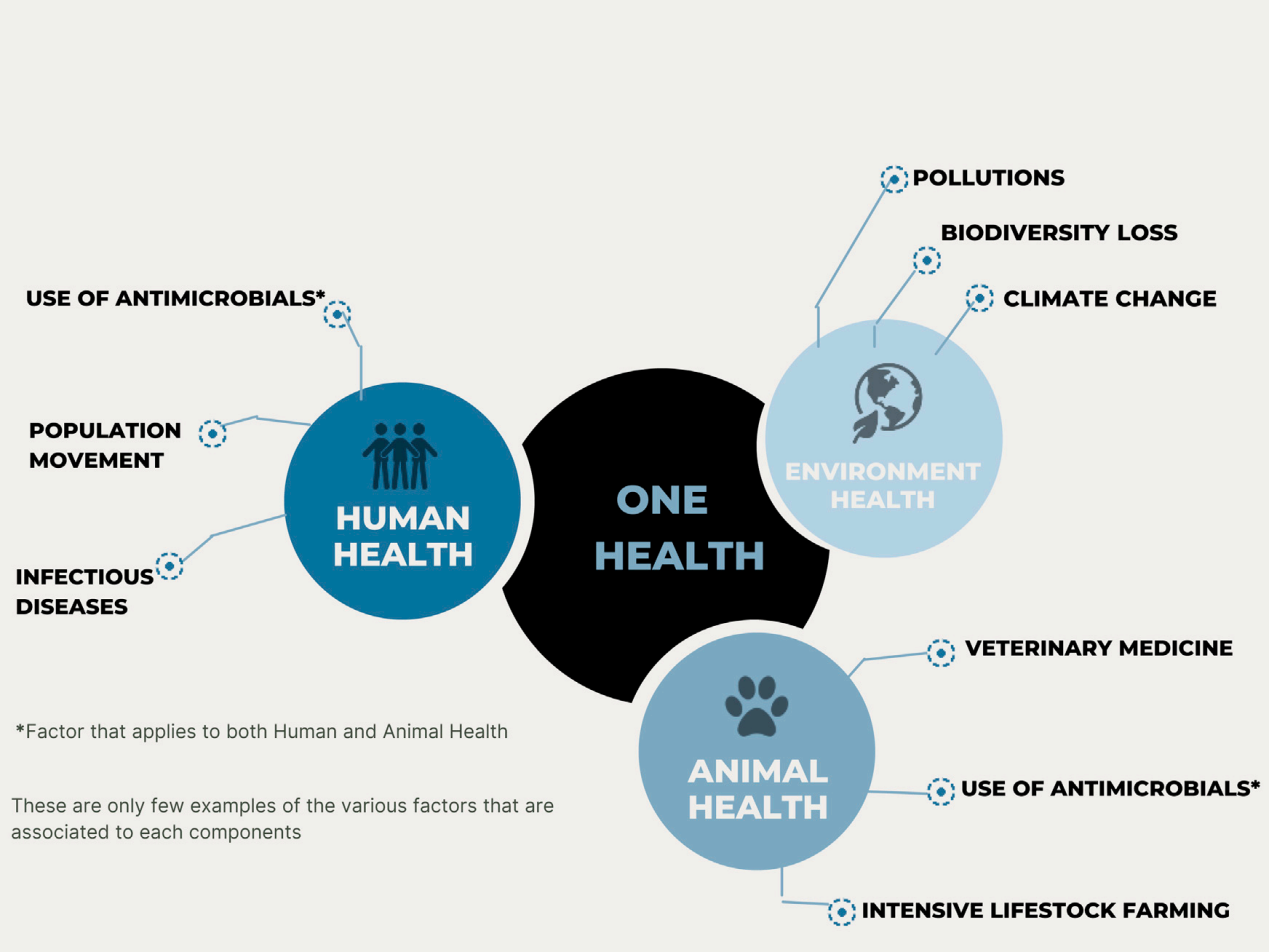
Illustration representing the concept of One Health Approach.

AMR is an emerging issue where a unified global approach is required. “*One Health*” embraces the concept that there is a clear connection between the health of both humans and animals and the shared surrounding environment as shown (Figure 3) [39]. It was evidenced that humans and animals can share the same bacteria, diseases and more importantly, the sharing of the same antibiotics to treat infectious diseases in animals, as well as in humans [40]. With all aspects considered, AMR has emerged as one of the most prominent “*One Health*” issues, since AMR has the ability to spread rapidly across the population as well as in the food chain, healthcare settings and the environment, thereby making it more challenging to manage many infectious diseases in both humans and animals [40].

The “*One Health Concept*” is a multisectoral approach, where combined efforts from all stakeholders are required to successfully engage in this program. The WHO has worked in close collaboration with the Food and Agriculture Organization of the United Nations (FAO) and the World Organization of Animal Health (OIE) to ensure effective actions are taken from all sectors in reducing the risks of AMR from this approach [1].

A growing number of studies have shifted their attention from human factors to more serious contributing AMR factors arising from animal aspects. Earlier research indicated that 73% of all antibiotics used worldwide are used in farm animals, largely for food production [14]. This would become a serious issue if continuously progressing without being controlled. Van Boeckel et al. have proposed user fees and regulatory caps on veterinary use of antimicrobials as a means to reduce animal consumption of antimicrobials [41].

Additionally, the agriculture sector is taking a proactive role in ensuring the responsible use of veterinary medicines, through the development of various antimicrobial stewardship programmes. RUMA (Responsible Use of Medicines in Agriculture Alliance) encourages innovative and proactive efforts to improve the responsible use of veterinary medicines while ensuring optimum animal health and welfare [42].

Additionally, the idea of “*One Health*” emphasises the interdependence of human and animal health [43, 44]. Therefore, guidelines on veterinary antimicrobials should be included to address the possible risk associated with the use of these medications, including the development of AMR. This information should not be restricted to providing human antimicrobials medications only [43]. This approach will further raise people’s awareness of the current issue of AMR.

The implication of the “*One Health Approach*” on AMR is rather challenging to be successfully conducted in LMICs compared to HICs. LMICs still remain to be the most affected by AMR as to facing more serious challenges that do not apply in HICs such as poverty, corruption and healthcare systems leading to a more disease-prone environment such as lack of clean water access and sanitation issue and even the accessibility of antimicrobials [45]. A previous study has also noted a further concern in the farming sectors of LMICs where the only option available to farmers to combat widespread bacterial infection in an animal is by the use of antibiotics due to lack of access to professional veterinarians and support systems [46]. However, AMR is still most closely monitored in HICs, despite the fact that many of the most modified resistant strains of bacteria are particularly transmitted in LMICs [47]. This demonstrates a development gap in these nations’ healthcare systems and AMR regulations which need to be addressed.

Several approaches can strengthen this “*One Health Approach*,” especially in LMICs, by possibly introducing antimicrobial-sensitive intervention such as clean water access, this approach would be more cost-effective and sustainable in some resources limited LMICs.

### Global Action Plan (GAP) and Global Antimicrobial Resistance and Use Surveillance System (GLASS)

In addition to the “*One Health Approach*,” in 2015, the WHO launched an AMR-related management program known as the Global Action Plan focusing on AMR (GAP-AMR). This plan aims to ensure that infectious disease can always be successfully treated and prevented by preserving antimicrobials in a responsible manner with appropriate accessibility and quality checks [48].

Five main objectives were established to fulfil this goal, one of which is to enhance the efficacy of antimicrobial medication use for both human and animal health, this would require a good regulatory system, respectively [48]. Some of the most recognisable drug regulatory agencies such as the Food and Drug Administration (FDA) in the United States, and the Health Products Regulatory Authority (HPRA) in Ireland, both of which regulate medicine for human and animal health. In comparison, the UK has a separate system for humans and animals, the Medicines and Healthcare products Regulatory Agency (MHRA) for human health and the Veterinary Medicines Directorate (VMD) for animal health.

It is expected that countries would introduce their national plans in AMR in order to reach the goals of the overall Global Action Plan. According to the WHO, 18 countries and territories have established multi-stakeholder national action plans on AMR while six countries are in the final stages of creating national action plans [49].

The other approach launched was the Global Antimicrobial Resistance and Use Surveillance System (GLASS). The aim of this program is to focus on promoting surveillance of AMR globally and determining the causes of AMR. This would involve providing advice and guidance to support nations to implement remedial measures as necessary [50].

## Antimicrobial Resistance and the COVID-19 Pandemic

The occurrence of the COVID-19 pandemic in 2020 created an unprecedented scenario for the treatment of suspected or proven bacterial infections with antibiotics. The changing dynamic in the movement of patients within healthcare (community and hospital), largely driven by the patients’ SARS CoV-2 status, combined with reduced diagnostic ability due to redeployment of laboratory staff to SARS CoV-2 testing, resulted in a temporary redefining of “Best Practice” antimicrobial stewardship programmes, due to pandemic-related constraints. A recent study investigating global antibiotic use during the pandemic, through examination of pharmaceutical sales data from 71 countries during the years, 2020–2022, showed that sales of cephalosporins, penicillins, macrolides, and tetracyclines decreased dramatically during April–May 2020, afterwhich there was a gradual rise to nearly pre-pandemic rates through May 2022 [51]. This study calculated that a 10% increase in monthly COVID-19 cases was associated with 0.2%–0.3% higher sales of cephalosporins, 0.2%–0.3% higher sales of penicillins, 0.4%–0.6% higher sales of macrolides, and 0.3% higher sales of all four antibiotics combined per 1,000 people. Geographically, a 10% increase in monthly COVID-19 cases was associated with 0.8%, 1.3%, and 1.5% higher macrolides sales in Europe, North America, and Africa, respectively. The U.S. Centers for Disease Control and Prevention estimates that antibiotic resistance in the United States increased 15% during 2019–2020, leading to 29,400 additional deaths, of which 40% were from hospital-acquired infections [52].

## Antimicrobial Resistance and the United Nations Sustainable Development Goals

**FIGURE 4:**
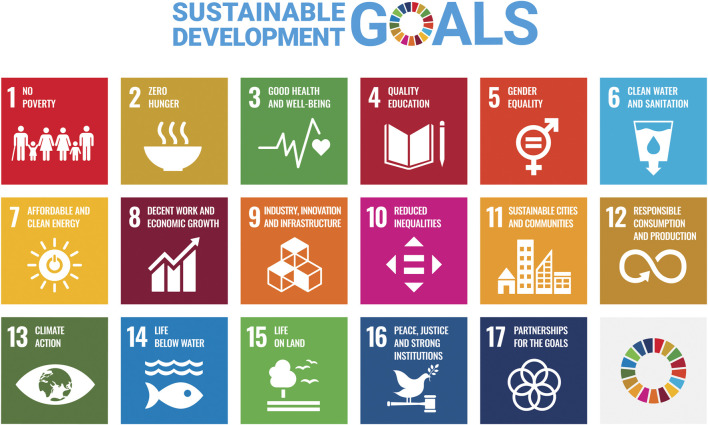
The 17 sustainable development goals of the United Nations (Source [54]).

**FIGURE 5:**
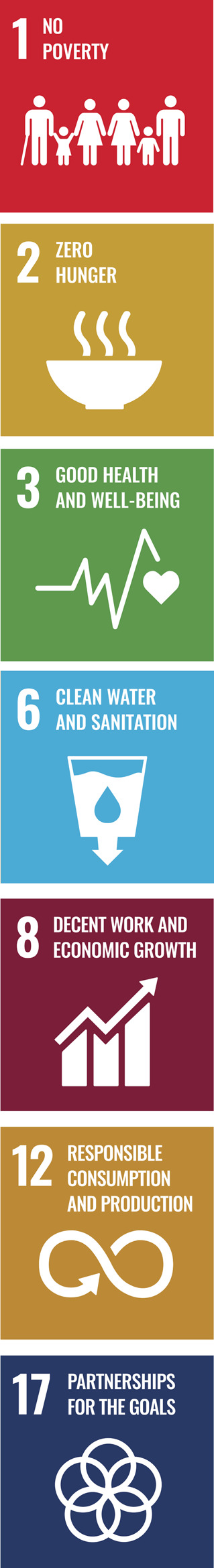
The seven United Nations Sustainable Development Goals (SDGs) affected by antimicrobial resistance.

As part of the 2030 Agenda for Sustainable Development, the United Nations has published 17 Sustainable Development Goals (SDGs) including many aspects of societal and human interactions, as illustrated in Figure 4 [53]. One SDG is SDG3–“Good Health and Wellbeing.” Within this, AMR has a named specific inclusion, with indicator 3.d.2, namely, “*Percentage of bloodstream infections due to selected antimicrobial-resistant organisms*.” The WHO have indicated that AMR impacts seven of the 17 SDGs, as detailed in Figure 5 and these are futher discussed by Jasovský et al. [55], particularly in terms of environmental, social, and economic targets in the SDG framework.

### The Role of Biomedical Science in Helping Combat AMR

The clinical microbiology laboratory, as well as biomedical scientists, have an important role to play in combatting AMR, both locally and internationally. Diagnostics play a vital role in adding value in terms of 1) laboratory surveillance of AMR epidemiology, through monitoring, 2) providing antimicrobial susceptibility results for clinically significant pathogens, to help avoid empirical antibiotic prescribing, but rather evidence-led antibiotic selection, 3) helping to determine if the aetiology of clinical infections are viral or bacterial, thereby helping decide the potential value of intervening with antibiotics, 4) providing innovative techniques to robustly allow more rapid turnaround times for reporting of antimicrobial susceptibility results to antibiotic prescribers, so that good antimicrobial stewardship practices are promoted and 5) allowing the elucidation of novel mechanisms of antimicrobial resistance, through whole genome sequencing techniques. Recent advances in biomedical science innovative has allowed the development of total laboratory automation for the rapid detection and identification of pathogens, as well as for the determination of their antimicrobial susceptibility [56]. Additionally, IT capacity in terms of having sufficient laboratory informatics capacity, including data management systems, is important for effective laboratory surveillance of AMR, as well as the interface to share AMR susceptibility data, on a global basis [57].

AMR places new challenges for the clinical diagnostic laboratory. Our group recently reported that traditional microbiological agar formulations may become less efficient in their selective properties due to breakthrough of AMR organisms, where current concentrations of antibiotics have become ineffective in containing breakthrough from AMR organisms [58]. The emergence of AMR now threatens the ability to detect pathogens using traditional selective microbiological media, without a radical rethink and reformulation of many of these selective agars [58].

One example of this is the growing problem of highly resistant organisms, especially *Pseudomonas aeruginosa*, overgrowing agar cultures designed for the detection of *Mycobacterium tuberculosis* [59].

## Conclusion

Antimicrobial resistance continues to be a major global public health dilemma of the 21st century. Already this topic is receiving substantial political input from the G7 countries and continues to be on the agenda of numerous political conferences. Adoption of mitigating strategies are beginning to see positive results, with tangible outcomes (see Table 1). However, the consequences of failure to adequately address AMR are profound, with estimations of a return to the pre-antibiotic era, where everyday infections relating to childbirth, surgery and open fractured limbs could be potentially life-threatening. AMR itself represents a microcosm of factors, including social anthropology, civil unrest/war, diasporas, ethnic displacement, political systems, healthcare, economics, societal behaviour both at a population and individual level, health literacy, geoclimatic events, global travel and pharmaceutical innovation and investment, thus finding a solution that adequately addresses AMR and which helps stem further AMR emergence is complicated. Success will involve individuals, communities and nations all working together to ensure that the world continues to possess a sufficient armamentarium of effective antimicrobials that will sustain human and animal health, both now and in the future.

**TABLE 1:** Antimicrobial Resistance (AMR) reduction strategies and related outcomes.

Antimicrobial resistance theme	Description	Outcome
AMR Global/National Action Plans	WHO Framework of interventions to slow the emergence and reduce the spread of AMR	Many countries have now developed and published their national AMR Action plans. Certain countries (e.g., Japan, Tanzania, China) have now published their second national plan on AMR.
AMR Surveillance	WHO Global Antimicrobial Resistance and Use Surveillance System (GLASS)	The ongoing collection of antimicrobial susceptibility data on human and veterinary pathogens.
	Study for Monitoring Antimicrobial Resistance Trends (SMART)	Surveillance data is available through reports in scientific and medical journals, as well as freely available on hosts’ websites, e.g., EARS-net (https://www.ecdc.europa.eu/en/about-us/networks/disease-networks-and-laboratory-networks/ears-net-data)
	U.S. National Antimicrobial Resistance Monitoring System (NARMS)	AMR surveillance helps informs health policy.
	European Antimicrobial Resistance Surveillance Network (EARS-Net)	AMR surveillance data allows for examination of temporal and spatial trends of AMR locally, nationally and internationally.
AMR Diagnostics	Development of commercial laboratory diagnostic kits (phenotypic and molecular) to test for AMR bacteria or antimicrobial resistance markers/determinants in clinical, veterinary, food/water and environmental isolates	Investment by biotechnology companies into developing commercially available kits has allowed the widespread adoption of novel AMR diagnostics for AMR detection either in realtime—for the clinical management of patients or for AMR surveillance purposes.
AMR Public Health Messaging	World Antimicrobial Awareness Week (WAAW)	WAAW is a global campaign that aims to raise awareness of antimicrobial resistance worldwide and encourage best practices among the general public, health workers and policymakers to slow the development and spread of drug-resistant infections [60].
	British Society for Antimicrobial Chemotherapy (BSAC)	“*Stop Superbugs*” campaign to raise public awareness and support antibiotic health literacy initiatives [61].
	US Centers for Disease Control and Prevention	“*Combatting Antimicrobial Resistance*.” A portfolio of guidance, epidemiology and communication resources to support AMR public awareness [62].
AMR Antimicrobial Stewardship	An organisational or healthcare-system-wide approach to promote and monitor the judicious use of antimicrobials to preserve their future effectiveness [63].	Enhancement of the role of pharmacists to establish Antimicrobial Pharmacists, to support Antimicrobial Stewardship programmes
		Establishment of Antimicrobial Stewardship Committees in healthcare institutions to develop local antimicrobial policies.
		Establishment of Antimicrobial Stewardship policies in animal medicine (National Office of Animal Health; NOAH) and RUMA (Responsible Use of Medicines in Agriculture Alliance) [42]

## References

[1] World Health Organization. Antimicrobial Resistance (2021). Available from: https://www.who.int/news-room/fact-sheets/detail/antimicrobial-resistance (Accessed March 21, 2023).

[2] Prestinaci F Pezzotti P Pantosti A. Antimicrobial Resistance: a Global Multifaceted Phenomenon. Pathog Glob Health (2015) 109(7):309–18. 10.1179/2047773215Y.0000000030 26343252 PMC4768623

[3] Llor C Bjerrum L. Antimicrobial Resistance: Risk Associated with Antibiotic Overuse and Initiatives to Reduce the Problem. Ther Advdrug Saf (2014) 5(6):229–41. 10.1177/2042098614554919 PMC4232501 25436105

[4] Founou RC Blocker AJ Noubom M Tsayem C Choukem SP Dongen MV The COVID-19 Pandemic: a Threat to Antimicrobial Resistance Containment. Future Sci (2021) 7(8):FSO736. 10.2144/fsoa-2021-0012 PMC8204817 34290883

[5] O’Neill J. Review on Antimicrobial Resistance. Tackling Drug-Resistant Infections Globally (2016). Available from: https://amr-review.org/sites/default/files/160525_Final%20paper_with%20cover.pdf (Accessed March 21, 2023).

[6] World Health Organization. Monitoring and Evaluation of the Global Action Plan on Antimicrobial Resistance (2019). Available from: https://www.who.int/publications/i/item/monitoring-and-evaluation-of-the-global-action-plan-on-antimicrobial-resistance (Accessed March 21, 2023).

[7] World Health Organization. Comprehensive Review of the WHO Global Action Plan on Antimicrobial Resistance Volume 1: Report WHO Evaluation Office (2021). Available from: https://cdn.who.int/media/docs/default-source/documents/about-us/evaluation/gap-amr-final-report-v2.pdf?sfvrsn=1db7e8b0_1&download=true (Accessed March 21, 2023).

[8] Mostafa A Abdelzaher A Rashed S AlKhawaga SI Afifi SK AbdelAlim S Is Health Literacy Associated with Antibiotic Use, Knowledge and Awareness of Antimicrobial Resistance Among Non-medical university Students in Egypt? A Cross-Sectional Study. BMJ Open (2021) 11(3):e046453. 10.1136/bmjopen-2020-046453 PMC8098941 33649060

[9] Aminov RI. A Brief History of the Antibiotic Era: Lessons Learned and Challenges for the Future. Front Microbiol (2010) 1(134):134. 10.3389/fmicb.2010.00134 21687759 PMC3109405

[10] Hutchings MI Truman AW Wilkinson B. Antibiotics: Past, Present and Future. Curr Opin Microbiol (2019) 51(1):72–80. 10.1016/j.mib.2019.10.008 31733401

[11] Lobanovska M Pilla G. Penicillin’s Discovery and Antibiotic Resistance: Lessons for the Future? Yale J Biol Med (2017) 90(1):135–45. 28356901 PMC5369031

[12] Centers for Disease Control and Prevention. Antibiotic Resistance Threats in the United States (2013). Available from: https://www.cdc.gov/drugresistance/pdf/ar-threats-2013-508.pdf (Accessed March 21, 2023).

[13] Zaman SB Hussain MA Nye R Mehta V Mamun KT Hossain N. A Review on Antibiotic Resistance: Alarm bells Are Ringing. Cureus (2017) 9(6):e1403. 10.7759/cureus.1403 28852600 PMC5573035

[14] Van Boeckel TP Pires J Silvester R Zhao C Song J Criscuolo NG Global Trends in Antimicrobial Resistance in Animals in Low- and Middle-Income Countries. Science (2019) 365(6459):eaaw1944. 10.1126/science.aaw1944 31604207

[15] McKernan C Benson T Farrell S Dean M. Antimicrobial Use in Agriculture: Critical Review of the Factors Influencing Behaviour. JAC Antimicrob Resist (2021) 3(4):dlab178. 10.1093/jacamr/dlab178 34859222 PMC8634307

[16] Pokharel S Shrestha P Adhikari B. Antimicrobial Use in Food Animals and Human Health: Time to Implement 'One Health' Approach. Antimicrob Resist Infect Control (2020) 9(1):181. 10.1186/s13756-020-00847-x 33160396 PMC7648983

[17] Centers for Disease Control and Prevention. Zoonotic Diseases. Centers for Disease Control and Prevention (2021). Available from: https://www.cdc.gov/onehealth/basics/zoonotic-diseases.html (Accessed March 21, 2023).

[18] Gilbert W Thomas LF Coyne L Rushton J. Review: Mitigating the Risks Posed by Intensification in Livestock Production: the Examples of Antimicrobial Resistance and Zoonoses. Animal (2021) 15(2):100123. 10.1016/j.animal.2020.100123 33573940

[19] Liu Y-Y Wang Y Walsh TR Yi L-X Zhang R Spencer J Emergence of Plasmid-Mediated Colistin Resistance Mechanism MCR-1 in Animals and Human Beings in China: a Microbiological and Molecular Biological Study. Lancet Infect Dis (2016) 16(2):161–8. 10.1016/S1473-3099(15)00424-7 26603172

[20] Centers for Disease Control and Prevention. Newly Reported Gene, Mcr-1, Threatens Last-Resort Antibiotics Centers for Disease Control and Prevention (2019). Available from: https://www.cdc.gov/drugresistance/solutions-initiative/stories/gene-reported-mcr.html (Accessed March 21, 2023).

[21] Public Health England. Antibiotic Use and Resistance: What the Public Know (2020). Available from: https://www.gov.uk/government/publications/antibiotic-use-and-resistance-what-the-public-know (Accessed March 21, 2023).

[22] Bhardwaj K Shenoy MS Baliga S Unnikrishnan B Baliga BS. Knowledge, Attitude, and Practices Related to Antibiotic Use and Resistance Among the General Public of Coastal South Karnataka, India – A Cross-Sectional Survey. Clin Epidemiol Glob Health (2021) 11:100717. 10.1016/j.cegh.2021.100717

[23] Torumkuney D Poojary A Shenoy B Nijhara P Dalal K Manenzhe R. Country Data on AMR in India in the Context of Community-Acquired Respiratory Tract Infections: Links between Antibiotic Susceptibility, Local and International Antibiotic Prescribing Guidelines, Access to Medicine and Clinical Outcome. J Antimicrobiol Chemo (2022) 77(1):i10–7. 10.1093/jac/dkac212 PMC9445854 36065726

[24] Public Health England. New Antibiotic-Resistant Infections Rise to 178 Per Day in England (2020). Available from: https://www.gov.uk/government/news/new-antibiotic-resistant-infections-rise-to-178-per-day-in-england (Accessed March 21, 2023).

[25] European Centre for Disease Prevention and Control. Antimicrobial Resistance Surveillance in Europe 2022 - 2020 Data (2022). Available from: https://www.ecdc.europa.eu/en/publications-data/antimicrobial-resistance-surveillance-europe-2022-2020-data (Accessed March 21, 2023).

[26] Murray CJ Ikuta KS Sharara F Swetschinski L Aguilar GR Gray A Global burden of Bacterial Antimicrobial Resistance in 2019: a Systematic Analysis. Lancet (2022) 399(10325):629–55. 10.1016/S0140-6736(21)02724-0 35065702 PMC8841637

[27] Sifri Z Chokshi A Cennimo D Horng H. Global Contributors to Antibiotic Resistance. J Glab Infect Dis (2019) 11(1):36–42. 10.4103/jgid.jgid_110_18 PMC6380099 30814834

[28] Do NTT Vu HTL Nguyen CTK Punpuing S Khan WA Gyapong M Community-based Antibiotic Access and Use in Six Low-Income and Middle-Income Countries: a Mixed-Method Approach. Lancet Glob Health (2021) 9(5):e610–e619. 10.1016/S2214-109X(21)00024-3 33713630 PMC8050200

[29] Tiseo K Huber L Gilbert M Robinson TP Van Boeckel TP. Global Trends in Antimicrobial Use in Food Animals from 2017 to 2030. Antibiotics (2020) 9(12):918. 10.3390/antibiotics9120918 33348801 PMC7766021

[30] Browne AJ Chipeta MG Haines-Woodhouse G Kumaran EPA Hamadani BHK Zaraa S Global Antibiotic Consumption and Usage in Humans, 2000–18: a Spatial Modelling Study. Lancet Planet Health (2021) 5(12):e893–904. 10.1016/S2542-5196(21)00280-1 34774223 PMC8654683

[31] World Health Organization. WHO Report on Surveillance of Antibiotic Consumption (2019). Available from: https://www.who.int/publications/i/item/who-report-on-surveillance-of-antibiotic-consumption (Accessed March 21, 2023).

[32] ECDC, EFSA, EMA, OECD. Antimicrobial Resistance in the EU/EEA - A One Health Response. Solna, Sweden: European Centre for Disease Prevention and Control (2022). Available from: https://www.ecdc.europa.eu/en/publications-data/antimicrobial-resistance-eueea-one-health-response (Accessed March 21, 2023).

[33] Mladenovic-Antic S Kocic B Velickovic-Radovanovic R Dinic M Petrovic J Randjelovic G Correlation between Antimicrobial Consumption and Antimicrobial Resistance of *Pseudomonas aeruginosa* in a Hospital Setting: a 10-year Study. J Clin Pharm Ther (2016) 41(5):532–7. 10.1111/jcpt.12432 27511808

[34] Liang C Zhang X Zhou L Meng G Zhong L Peng P. Trends and Correlation between Antibacterial Consumption and Carbapenem Resistance in Gram-Negative Bacteria in a Tertiary Hospital in China from 2012 to 2019. BMC Infect Dis (2021) 21(1):444. 10.1186/s12879-021-06140-5 34001022 PMC8130264

[35] Kim B Kim Y Hwang H Kim J Kim S-W Bae I-G Trends and Correlation between Antibiotic Usage and Resistance Pattern Among Hospitalized Patients at university Hospitals in Korea, 2004 to 2012: A Nationwide Multicenter Study. Medicine (2018) 97(51):e13719. 10.1097/MD.0000000000013719 30572507 PMC6320075

[36] OneHealthTrust. ResistanceMap: Antibiotic Use (2023). Available from: https://resistancemap.onehealthtrust.org/AntibioticUse.php (Accessed March 21, 2023).

[37] Gyssens IC Wertheim HF. Editorial: Antimicrobial Stewardship in Low- and Middle-Income Countries. Front Public Health (2020) 8:617000. 10.3389/fpubh.2020.617000 33344409 PMC7738324

[38] Fink G D’Acremont V Leslie HH Cohen J. Antibiotic Exposure Among Children Younger Than 5 Years in Low-Income and Middle-Income Countries: a Cross-Sectional Study of Nationally Representative Facility-Based and Household-Based Surveys. Lancet Infect Dis (2019) 20(2):179–87. 10.1016/S1473-3099(19)30572-9 31843383

[39] Soto S. One Health: How to Achieve Optimal Health for People, Animals and Our Planet (2021). Available from: https://www.isglobal.org/en/healthisglobal/-/custom-blog-portlet/one-health-una-sola-salud-o-como-lograr-a-la-vez-una-salud-optima-para-las-personas-los-animales-y-nuestro-planeta/90586/0 (Accessed March 21, 2023).

[40] Centers for Disease Control and Prevention. One Health Basics (2018). Available from: https://www.cdc.gov/onehealth/basics/index.html (Accessed March 21, 2023).

[41] Van Boeckel TP Glennon EE Chen D Gilbert M Robinson TP Grenfell BT Reducing Antimicrobial Use in Food Animals. Science (2017) 357(6358):1350–2. 10.1126/science.aao1495 28963240 PMC6510296

[42] RUMA. Responsible Use of Medicines in Agriculture Alliance (2023). Available from: https://www.ruma.org.uk/ (Accessed May 16, 2023).

[43] EUR-Lex (2019). Regulation (EU) 2019/6 of the european parliament and of the council of 11 december 2018 on veterinary medicinal products and repealing directive 2001/82/EC (Text with EEA relevance). Available from: https://eur-lex.europa.eu/legal-content/EN/TXT/PDF/?uri=CELEX:32019R0006 (Accessed March 21, 2023).

[44] Widmer AF. Emerging Antibiotic Resistance: Why We Need New Antibiotics. Swiss Med Wkly (2022) 152:40032. 10.57187/smw.2022.40032 36351303

[45] Pokharel S Raut S Adhikari B. Tackling Antimicrobial Resistance in Low-Income and Middle-Income Countries. BMJ Glob Health (2019) 4(6):e002104. 10.1136/bmjgh-2019-002104 PMC6861125 31799007

[46] Robinson TP Bu DP Carrique-Mas J Fèvre EM Gilbert M Grace D Antibiotic Resistance Is the Quintessential One Health Issue. Trans R Soc Trop Med Hyg (2016) 110(7):377–80. 10.1093/trstmh/trw048 27475987 PMC4975175

[47] Ikhimiukor OO Odih EE Donado-Godoy P Okeke IN. A Bottom-Up View of Antimicrobial Resistance Transmission in Developing Countries. Nat Microbiol (2022) 7(6):757–65. 10.1038/s41564-022-01124-w 35637328

[48] World Health Organization. Global Action Plan on Antimicrobial Resistance (2016). Available from: https://www.who.int/publications/i/item/9789241509763 (Accessed March 21, 2023). 10.7196/samj.9644 26242647

[49] World Health Organization. Tackling Antimicrobial Resistance (2022). Available from: https://www.who.int/westernpacific/activities/tackling-antimicrobial-resistance (Accessed March 21, 2023).

[50] World Health Organization. Global Antimicrobial Resistance and Use Surveillance System (GLASS) (2021). Available from: https://www.who.int/initiatives/glass (Accessed June 22, 2023).

[51] Nandi A Pecetta S Bloom DE. Global Antibiotic Use during the COVID-19 Pandemic: Analysis of Pharmaceutical Sales Data from 71 Countries, 2020-2022. EClinicalMedicine (2023) 57:101848. 10.1016/j.eclinm.2023.101848 36776504 PMC9900305

[52] Centers for Disease Control and Prevention. U.S. Department of Health and Human Services, Atlanta GA. COVID-19: U.S. Impact on Antimicrobial Resistance, Special Report 2022 (2022). Available from: https://www.cdc.gov/drugresistance/pdf/covid19-impact-report-508.pdf (Accessed on May 10, 2023).

[53] United Nations. Department of Economic and Social Affairs. The 17 Goals (2023). Available from: https://sdgs.un.org/goals (Accessed May 10, 2023).

[54] Sustainable Development Goals: United Nations Department of Global Communications. Guidelines for the use of the SDG Logo Including the Colour Wheel, and 17 Icons (2020). Available from: https://www.un.org/sustainabledevelopment/wp-content/uploads/2019/01/SDG_Guidelines_AUG_2019_Final.pdf (Accessed June 23, 2023).

[55] Jasovský D Littmann J Zorzet A Cars O. Antimicrobial Resistance-A Threat to the World's Sustainable Development. Ups J Med Sci (2016) 121(3):159–64. 10.1080/03009734.2016.1195900 27416324 PMC4967260

[56] Cherkaoui A Schrenzel J. Total Laboratory Automation for Rapid Detection and Identification of Microorganisms and Their Antimicrobial Resistance Profiles. Front Cel Infect Microbiol (2022) 12:807668. 10.3389/fcimb.2022.807668 PMC8851030 35186794

[57] Turner P Rupali P Opintan JA Jaoko W Feasey NA Peacock SJ Laboratory Informatics Capacity for Effective Antimicrobial Resistance Surveillance in Resource-Limited Settings. Lancet Infect Dis (2021) 21(6):e170–e174. 10.1016/S1473-3099(20)30835-5 33865461

[58] Moore JE Millar BC. The Day the agar Stopped Working: what Emerging Antimicrobial Resistance (AMR) Means for Microbiology Laboratory Testing-Potential Effects on Infectious Disease Reporting. Clin Microbiol Infect (2020) 26(8):973–5. 10.1016/j.cmi.2020.04.035 32360776

[59] McClean M Stanley T Stanley S Maeda Y Goldsmith CE Shepherd R Identification and Characterization of Breakthrough Contaminants Associated with the Conventional Isolation of *Mycobacterium tuberculosis* . J Med Microbiol (2011) 60(9):1292–8. 10.1099/jmm.0.030619-0 21527550

[60] World Antimicrobial Awareness Week. World Health Organization (WHO) (2023). Available from: https://www.who.int/campaigns/world-antimicrobial-awareness-week (Accessed May 16, 2023).

[61] British Society for Antimicrobial Chemotherapy (BSAC). Stop Superbugs Campaign (2020). Available from https://bsac.org.uk/bsac-launches-stop-superbugs/ (Accessed May 16, 2023).

[62] Centers for Disease Control and Prevention. Combatting Antimicrobial Resistance, a Global Threat (2021). Available from https://www.cdc.gov/DrugResistance/ (Accessed May 16, 2023).

[63] Department of Health and Social Care and Public Health England. Antimicrobial Prescribing and Stewardship Competencies (2013). Available from: https://www.gov.uk/government/publications/antimicrobial-prescribing-and-stewardship-competencies (Accessed May 16, 2023).

